# Supplementary material for: map3k1 suppresses terminal differentiation of migratory eye progenitors in planarian regeneration
Source: bioRxiv. 2024 Oct 12:2024.10.11.617745. Preprint. [Version 1] doi: 10.1101/2024.10.11.617745 (PMC11483071; doi:10.1101/2024.10.11.617745)

bioRxiv preprint doi: https://doi.org/10.1101/2024.10.11.617745; this version posted October 12, 2024. The copyright holder for this preprint (which was not certified by peer review) is the author/funder, who has granted bioRxiv a license to display the preprint in perpetuity. It is made available under aCC-BY 4.0 International license.

Supplementary Figure Legends

Figure S1. (A) Domain structure of dd_Smed_v6_5198_0_1 (*map3k1)* containing a RING (E-value= 0.0255) domain, a serine/threonine kinase domain (E-value=1.16e-69) characteristic of MAPKs, and a transmembrane region. (B) Stacked bar graph quantifying the number of ectopic eyes in control (n=46) versus *map3k1(RNAi)* (n=48) animals over 8 weeks of RNAi showing that *map3k1* inhibition caused ectopic eyes to continue forming over time.

Figure S2. Homeostatic animals fixed after 4 weeks of RNAi were stained with *ChAT* to detect cholinergic neurons. *map3k1* RNAi did not increase *ChAT*$^{+}$ neuron staining, compared to the ectopic *ChAT*$^{+}$ brain branches that formed after *ndk* RNAi. n≥9. Scale bars, 300µm.

Figure S3. To test whether *map3k1* could control eye placement and/or a subset of head patterning, animals were fed with either control or *map3k1* dsRNA for 4 weeks before undergoing eye resections at different positions indicated by the cartoons. Individual live animals were imaged before, immediately after (post-resection), and 14 days post-surgical eye resection (14dpR) to track whether eye regeneration subsequently occurred. *map3k1(RNAi)* animals regenerated their original eyes at a high frequency (15/16). Ectopic eyes from these animals were also capable of regeneration, though at lower frequencies. Removal of either the anterior-most ectopic eyes (6/14 eyes regenerated, "1st ectopic") or the posterior-most ectopic eyes (4/7 eyes regenerated, "last ectopic") could result in regeneration from the original eye. Sample size, n≥7 animals in each condition.

bioRxiv preprint doi: https://doi.org/10.1101/2024.10.11.617745; this version posted October 12, 2024. The copyright holder for this preprint (which was not certified by peer review) is the author/funder, who has granted bioRxiv a license to display the preprint in perpetuity. It is made available under aCC-BY 4.0 International license.

Figure S4. (A) Single-cell RNA sequencing expression profile of *map3k1* during injury as published from a prior study (31). *map3k1* is expressed broadly and in many different tissues, but has enriched expressed in the muscle, neural, and gut clusters. (B) Maximum projection images of *map3k1* expression in homeostatic worms as detected by FISH show *map3k1* is expressed broadly throughout the body. Right panel, image showing *map3k1* expression in the head and low levels of *map3k1* expression in the eyes (arrows). Sample size, n=14 animals. Scale bar, 100µm. (C) Maximum projection images of *map3k1* and *opsin* expression in the eye show some expression of *map3k1* in *opsin* expressing cells (4/4 animals). Scale bar, 50µm. (D) Double-FISH detecting *ovo* and *map3k1* expression. Some ovo^{+} cells expressed low levels of *map3k1* (yellow arrow, 22/36 cells counted over 5 intact animals) while others did not have *map3k1* expression (white arrow, 14/36 cells over 5 animals). *map3k1* is also broadly expressed, so other unknown *map3k1*$^{+}$ but *ovo*$^{-}$ cells were identifiable (green arrow). Scale bar, 50µm.

bioRxiv preprint doi: https://doi.org/10.1101/2024.10.11.617745; this version posted October 12, 2024. The copyright holder for this preprint (which was not certified by peer review) is the author/funder, who has granted bioRxiv a license to display the preprint in perpetuity. It is made available under aCC-BY 4.0 International license.

939 **Table S1. Primer sequences**

Gene Name	dd_Smed_v6 number	Primer 1	Primer 2
map3k1	dd_Smed_v6_5198_0_1	tgtgcagaaaatcatggcct	tcataggctggctcaggagt
map2k6-1	dd_Smed_v6_1286_0_1	cagattcacccacgactcct	tcgatcaatgaagacgaccaa
map2k6-2	dd_Smed_v6_2106_0_1	cggaagttcaagttaatggtgag	caaagtcttacacgcgcaga
map2k4	dd_Smed_v6_3173_0_1	aagctggagctccaccgcgg	gggcgaattgggtaccggg
map2k1	dd_Smed_v6_3518_0_1	aatcataccgcgacaaagcc	cgttgaggagatttcgcagg
map2k7	dd_Smed_v6_3580_0_1	cgagtgaggtgtggatttgc	aatgacaacgaggctgagga
map2k3	dd_Smed_v6_4722_0_1	aagctggagctccaccgcgg	gggcgaattgggtaccggg
map2k2	dd_Smed_v6_5742_0_1	acttgaatcgacccgtcagt	caatagagtccgcggcaatg
map2k5	dd_Smed_v6_8370_0_1	aagctggagctccaccgcgg	gggcgaattgggtaccggg
wntA (wnt11-6)	dd_Smed_v6_4877_0_1	tgatgaagccttcgttgattt	tccaattgtgcaatgattcg
ndk	dd_Smed_v6_11285_0_1	gcacaataccgattgtcaaaccct	ggcttgataatggctaactggt
notum	dd_Smed_v6_2418_0_1	aaaatttctgaggatcgaaaaa	tgaagctagatttatgtgaaaaacca
sfrp1	dd_Smed_v6_13985_0_1	ttgaattcatggaaatgaccaa	aatcaatgaaatgttttgttgtga
ndl3	dd_Smed_v6_6604_0_1	ttattgacagtaggaaccaaagcc	atcctgaatcaagtcaacgcca
wnt1 (wntp-1)	dd_Smed_v6_28398_0_1	ggcgcttccatttgtttacc	gaagccctgataaaacaagca

bioRxiv preprint doi: https://doi.org/10.1101/2024.10.11.617745; this version posted October 12, 2024. The copyright holder for this preprint (which was not certified by peer review) is the author/funder, who has granted bioRxiv a license to display the preprint in perpetuity. It is made available under aCC-BY 4.0 International license.

fzd-4	dd_Smed_v6_11650_0_1	ggaatagcccaactcaccaa	tgccgaatttagttggaagc
wntP-2	dd_Smed_v6_7326_0_1	aacgcattagcattcataccg	aatgcaaattttggaatctga
p38a	dd_Smed_v6_1272_0_1	ctccatcatctgtctgtgagga	catctgtctgcctcgctaga
p38b	dd_Smed_v6_9252_0_1	gccacggtagtcaaggatct	tcatgcaatcccatattccatct
JNK	dd_Smed_v6_5924_0_1	gccattaaagcgtcattccg	tattggttccggtgcacaag

bioRxiv preprint doi: https://doi.org/10.1101/2024.10.11.617745; this version posted October 12, 2024. The copyright holder for this preprint (which was not certified by peer review) is the author/funder, who has granted bioRxiv a license to display the preprint in perpetuity. It is made available under aCC-BY 4.0 International license.

Table S2. Manual Cell Count Values for Nuclei Segmentation

**Image Name**	**True Positives (TP)**	**False Negative (FN)**	**False Positive (FP)**	**Total Cells (TP+FN+FP)**	**Jaccard Index**
Control(RNAi) Sample 1	9	5	0	14	0.642857
Control(RNAi) Sample 2	5	2	1	8	0.625
Control(RNAi) Sample 3	11	10	2	23	0.478261
Control(RNAi) Sample 4	10	11	1	22	0.454545
Control(RNAi) Sample 5	8	6	4	18	0.444444
Control(RNAi) Sample 6	10	2	3	15	0.666667
map3k1(RNAi) Sample 1	34	10	11	55	0.618182
map3k1(RNAi) Sample 2	19	15	0	34	0.558824
map3k1(RNAi) Sample 3	19	20	4	43	0.44186
map3k1(RNAi) Sample 4	33	11	14	58	0.568966
map3k1(RNAi) Sample 5	9	21	0	30	0.3
map3k1(RNAi) Sample 6	19	21	3	43	0.44186

bioRxiv preprint doi: https://doi.org/10.1101/2024.10.11.617745; this version posted October 12, 2024. The copyright holder for this preprint (which was not certified by peer review) is the author/funder, who has granted bioRxiv a license to display the preprint in perpetuity. It is made available under aCC-BY 4.0 International license.

Figure S1.

A

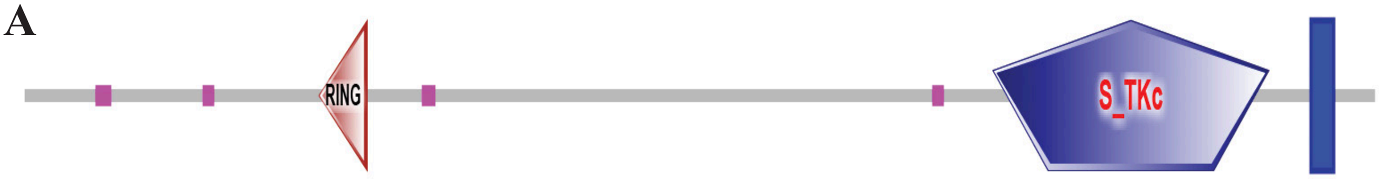

B

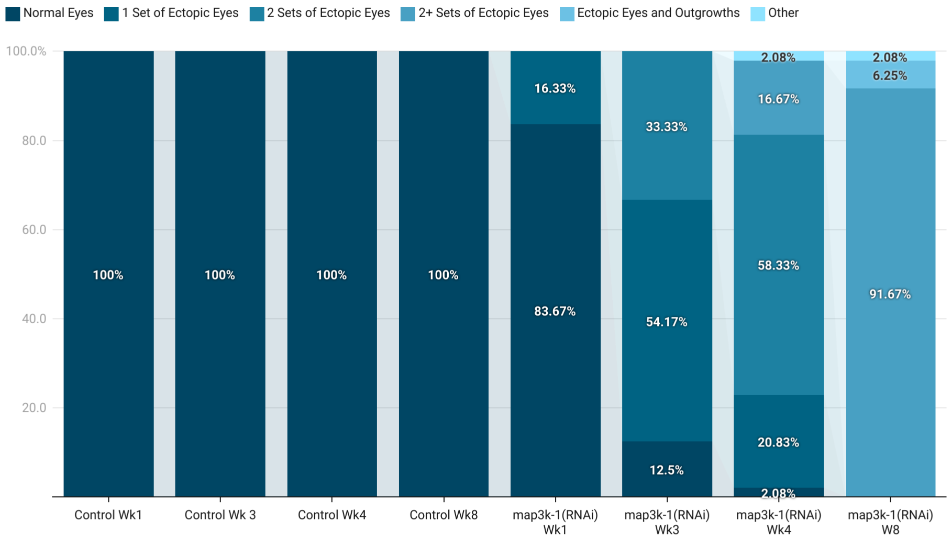

bioRxiv preprint doi: https://doi.org/10.1101/2024.10.11.617745; this version posted October 12, 2024. The copyright holder for this preprint (which was not certified by peer review) is the author/funder, who has granted bioRxiv a license to display the preprint in perpetuity. It is made available under aCC-BY 4.0 International license.

Figure S2.

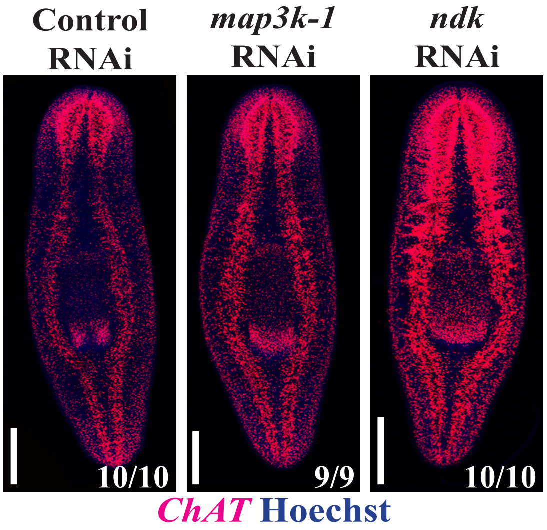

bioRxiv preprint doi: https://doi.org/10.1101/2024.10.11.617745; this version posted October 12, 2024. The copyright holder for this preprint (which was not certified by peer review) is the author/funder, who has granted bioRxiv a license to display the preprint in perpetuity. It is made available under aCC-BY 4.0 International license.

Figure S3.

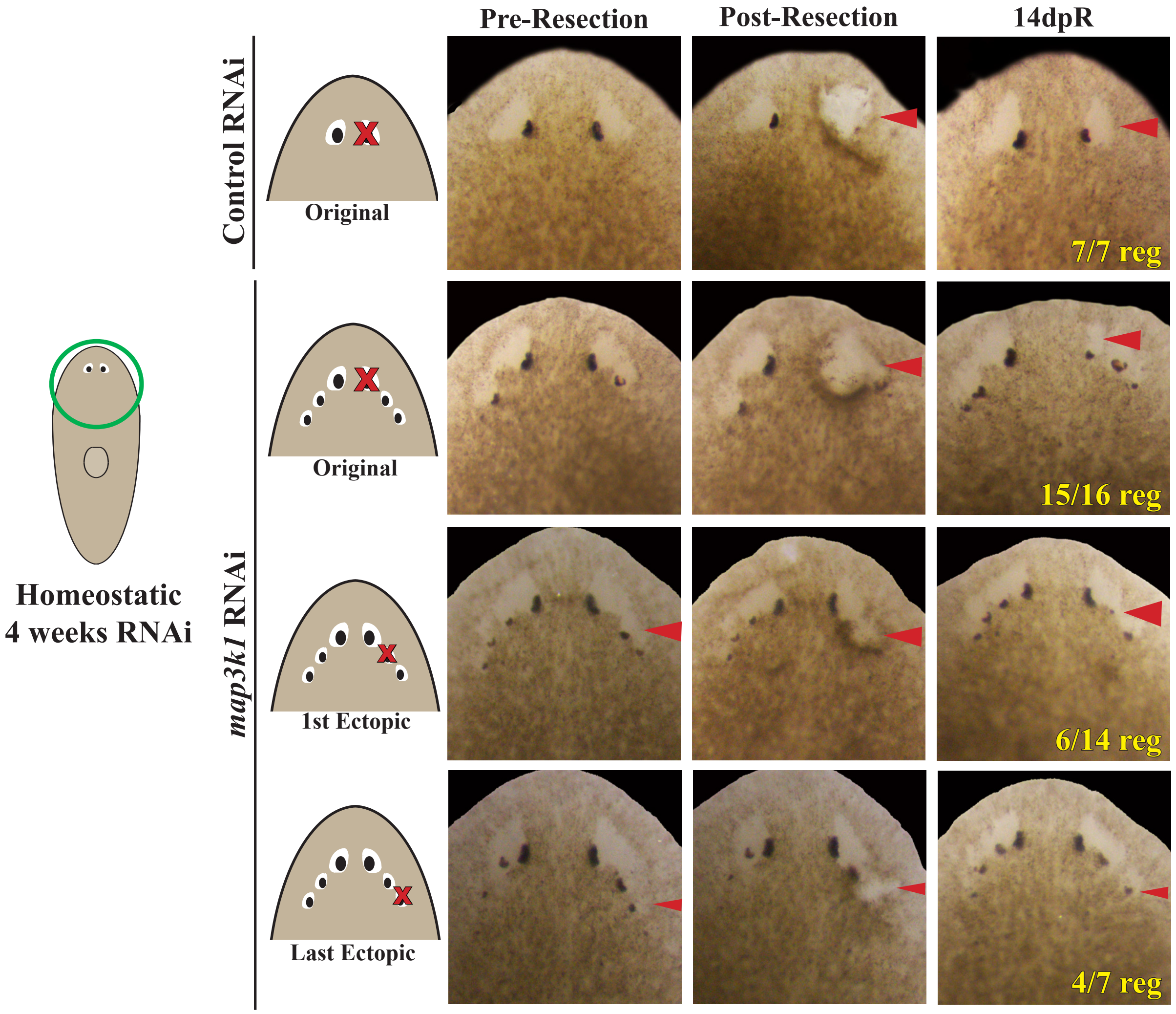

bioRxiv preprint doi: https://doi.org/10.1101/2024.10.11.617745; this version posted October 12, 2024. The copyright holder for this preprint (which was not certified by peer review) is the author/funder, who has granted bioRxiv a license to display the preprint in perpetuity. It is made available under aCC-BY 4.0 International license.

Figure S4.

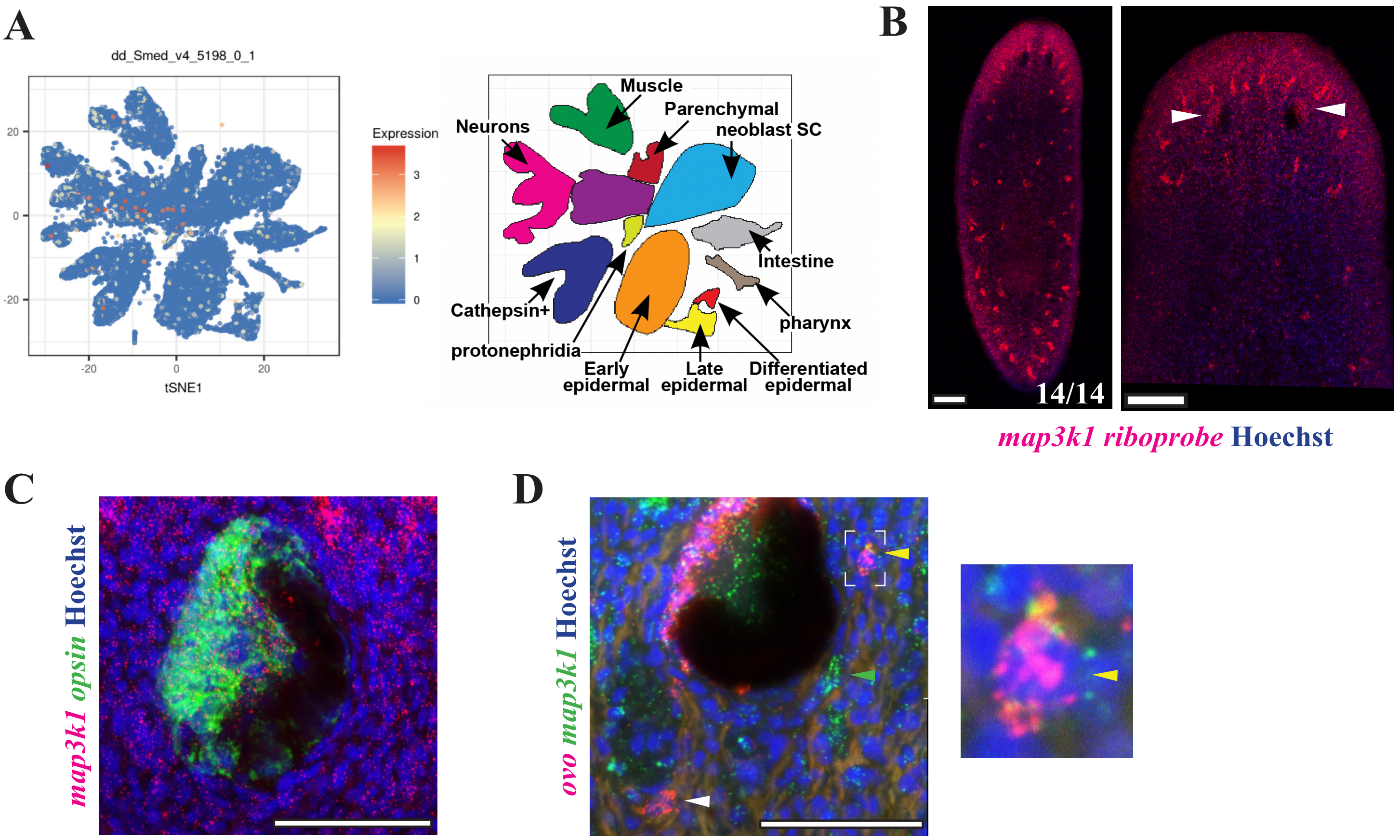

Supplement: Supplement 1 [file NIHPP2024.10.11.617745v1-supplement-1.pdf]
